# DNA repair helicases: from mechanistic understanding to therapeutic implications

**DOI:** 10.1093/narcan/zcaf034

**Published:** 2025-10-07

**Authors:** Vijay Menon, Susan E Gueble

**Affiliations:** Department of Therapeutic Radiology, Yale School of Medicine, New Haven, CT 06520-8040, United States; Department of Therapeutic Radiology, Yale School of Medicine, New Haven, CT 06520-8040, United States

## Abstract

The maintenance of genomic integrity is paramount for normal cell physiology and survival as well as avoidance of carcinogenesis. Cellular DNA is periodically subjected to a myriad of exogenous and endogenous threats and requires constant monitoring to limit genomic instability. To this end, cells possess an intricate DNA damage response and repair (DDR) module comprised of different classes of protein players. The DNA helicases, ATP-dependent enzymes that unwind the DNA double helix, are one such important class of proteins, which act as a linchpin between the recognition and resolution of DNA damage via facilitating various DNA repair processes. Dysfunction or absence of DDR helicase function is implicated in several human disorders including Bloom syndrome, Werner syndrome, Rothmund–Thomson syndrome, and Fanconi anemia. Somatic helicase mutations or dysregulation of helicase function can also contribute to cancer development, progression, and chemotherapy sensitivity, making helicases a promising target class for chemotherapeutic drug intervention. In addition, recent discoveries have identified some DDR helicases in novel synthetic lethal interactions. In this critical review, we will focus on human DNA helicases that are directly or indirectly involved in DDR with special emphasis on their mechanistic actions and clinical implications.

## Graphical Abstract

**Graphical Abstract:**
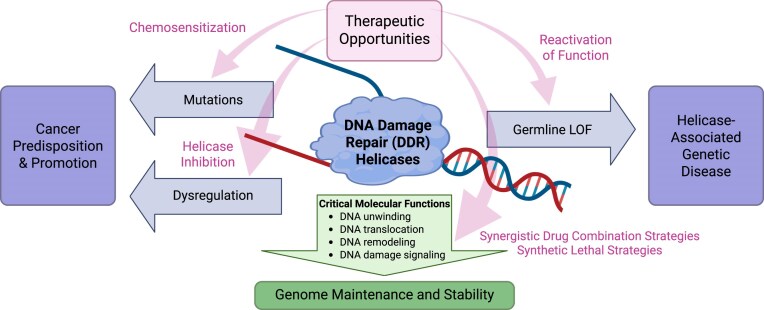

## Introduction

Genome integrity is critical to the survival of organisms and needs to be monitored and maintained consistently. Eukaryotic cells are constantly exposed to various forms of DNA damage, both internally as well as externally, including replication, oxidative, and genotoxic stress, and metabolic end-products. Unrepaired damage disrupts normal DNA transactions including replication and transcription leading to genomic instability. To circumvent these challenges, cells have evolved complex DNA damage response and repair (DDR) pathways that allow the timely detection of DNA lesions leading to extensive signal transduction and resolution of damage via the convergence of multiple DNA repair proteins and complexes. The highly regulated working of these pathways depends on the coordination between multiple cellular proteins and factors.

Helicases are ATP-dependent molecular engines that catalyze the unwinding of DNA and RNA, resolve replication- and transcription-associated secondary structures like G-quadruplexes and R-loops, and remove nucleoprotein complexes [1–3]. Through these molecular functions, helicases are intricately involved in most aspects of nucleic acid metabolism including DNA replication, transcription, translation, DNA repair, and telomere maintenance. A total of 95 helicases are encoded in the human genome, 31 DNA helicases and 64 RNA helicases [4]. They are divided into six different superfamilies, SF1-SF6, based on their activities, sequence homology, and structure [5–7].

**Figure 1.**
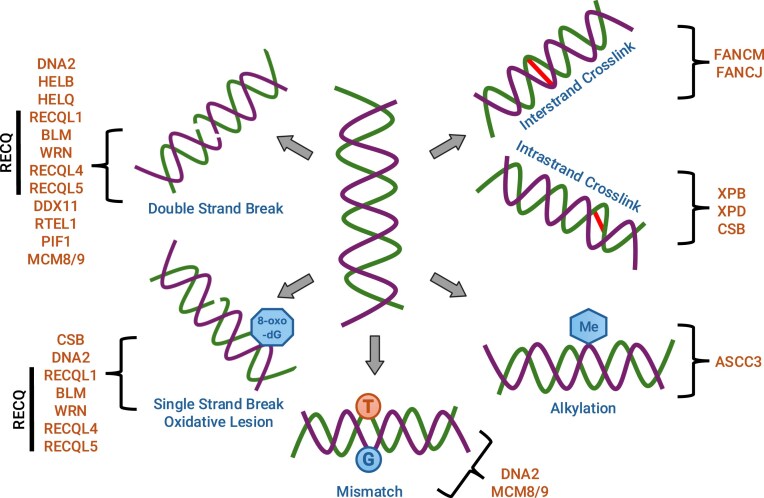
Involvement of DNA helicases in DDR pathways. DDR pathways are triggered as a result of damage to the DNA by a wide range of cellular or external factors. These pathways involve a myriad of cellular proteins of which helicases form an important group. The DDR helicases involved in the response to different types of DNA damage are depicted here. These include: DNA DSB-R (DNA2, HELB, HELQ, RECQL1, BLM, WRN, RECQL4, RECQL5, DDX11, RTEL1, PIF1, MCM8/9), DNA single strand break repair (SSB-R) or BER of oxidative lesions (CSB, DNA2, RECQL1, BLM, WRN, RECQL4, RECQL5), DNA ICL-R (FANCM, FANCJ), DNA intrastrand crosslink repair or NER of bulky lesions (XPB, XPD, CSB), DRR of alkylation damage (ASCC3), and DNA MMR (DNA2, MCM8/9). Figure references: [342–345].

Helicases have a direct pivotal role in several DNA repair pathways primarily including nucleotide excision repair (NER), DNA double strand break repair (DSB-R), and DNA interstrand crosslink repair (ICL-R) (Fig. 1). Additionally, helicases have been proposed to function in other DNA repair pathways including base excision repair (BER), mismatch repair (MMR), and direct reversal repair (DRR), with individual helicases often impinging on multiple DDR pathways. The highly characterized DDR-implicated helicases mainly include members of the helicase superfamily 2 (SF2), including RecQ family helicases (BLM, WRN, and RECQL1/4/5), iron-sulfur (Fe-S) cluster family helicases (DNA2, XPD, DDX11, FANCJ), and other SF2 helicases (XPB, CSB, FANCM, HELQ, ASCC3). A more limited number of members of the closely related superfamily 1 (SF1) helicases (DNA2, HELB, PIF1) and the evolutionarily distinct AAA + helicase family (MCM8, MCM9) are also implicated in DDR. The SF1 and SF2 helicases are characterized by highly structurally similar catalytic cores harboring two protein domains which resemble the folds of the RecA recombination protein. The functional differences between individual helicases arise from their nucleic acid substrate structure preference, polarity, regulation, and interactions with other DDR factors. In addition, noncanonical helicase activities (e.g. translocation without winding) can in some cases constitute critical functions.

Importantly, germline mutations in DDR helicases have been associated with various human genetic syndromes which display overlapping phenotypes predominantly characterized by cancer predisposition, premature aging, and developmental anomalies. In addition, as genomic instability is a hallmark feature of cancer cells and can be further induced with many chemotherapy agents, the critical role of DDR helicases in responding to these stressors offers promising opportunities for anticancer strategies. This review intends to provide a synopsis of the key molecular functions of DNA helicases in genome stability, emphasizing their established roles in DDR pathways. By deciphering the nexus between helicases and DDR-associated genome maintenance, we aim to illustrate the unique roles of these molecular “untanglers” and their potential as therapeutic targets.

## NER-associated helicases

NER is a major DNA repair pathway that resolves a wide range of bulky and helix-distorting lesions, including ultraviolet (UV)-induced cyclobutane pyrimidine dimers and 6–4 photoproducts, DNA adducts generated by environmental mutagens like polycyclic aromatic hydrocarbons, certain bulky oxidative lesions induced by reactive oxygen species, and intrastrand crosslinks formed by chemotherapeutic drugs like cisplatin [8–10]. NER can be divided into two sub-pathways: global genome NER (GG-NER) (reviewed extensively in [11]) occurring throughout the genome, and transcription coupled-NER (TC-NER) (reviewed extensively in [12]), occurring within transcribed regions of the genome. TC-NER is activated when RNA polymerase II (RNAPII) encounters a lesion during transcription elongation [13]). Cockayne Syndrome A (CSA) and Cockayne Syndrome B (CSB) sense the stalled RNAPII and promote the recruitment of transcription factor IIH (TFIIH) complex in conjunction with the UVSSA protein [14]. Other distinct lesion sensor proteins identify bulky lesions in GG-NER and also recruit TFIIH. Subsequent downstream NER factors incise the nucleotide lesion, fill the DNA gap, and ligate the DNA to complete the repair process. Out of the many protein players involved in the NER pathway, there are two main helicases involved in GG-NER, xeroderma pigmentosum B (XPB), and xeroderma pigmentosum D (XPD), both of which are components of TFIIH [15], and one helicase specific to TC-NER, CSB (Table 1).

**Table 1.** Helicases involved in the NER pathway

Helicase	Classification	Biochemical specificity	DDR function(s)	Genetic syndromes	Reported inhibitors	Additional DNA repair pathway involvement
XPB	SF2	3′–5′ DNA helicase	Component of TFIIH Performs initial DNA opening at NER site	XP, TTD, XPCS	Triptolide, spironolactone	-
XPD	SF2 (Fe-S cluster family)	5′–3′ DNA helicase	Component of TFIIH Unwinds double-stranded DNA (dsDNA) and recognizes NER lesion	XP, TTD, XPCS	-	-
CSB	SF2 (SWI/SNF family)	dsDNA translocase	Regulates TC-NER via recruitment of downstream factors to transcription-stalling lesions Modulates pathway choice in DSB-R, suppressing NHEJ and promoting HRR Recruits XRCC1 for BER Interacts with PARP1/2 in SSB-R	CS	-	BER, DSB-R, SSB-R

XPB, Xeroderma pigmentosum B; XPD, Xeroderma pigmentosum D; CSB, Cockayne syndrome B; SF2, Superfamily 2; TFIIH, Transcription factor IIH; TC-NER, Transcription coupled nucleotide excision repair; DSB-R, Double strand break repair; NHEJ, Nonhomologous end joining; HRR, Homologous recombination repair; XRCC1, X-ray repair cross-complementing protein 1; BER, Base excision repair; PARP1/2, Poly [ADP-ribose] polymerase 1/2; SSB-R, Single strand break repair; XP, Xeroderma pigmentosum; TTD, Trichothiodystrophy; XPCS, Combined XP and Cockayne syndrome; CS, Cockayne syndrome

### Xeroderma pigmentosum B (XPB/RAD25/ERCC3)

XPB/RAD25/ERCC3 is a 3′–5′ DNA helicase, belonging to the SF2 superfamily of helicases and a component of the TFIIH transcription complex (reviewed in [16]). Germline defects in XPB are implicated in patients with xeroderma pigmentosum (XP), trichothiodystrophy (TTD), or combined XP and Cockayne syndrome (XPCS), which are all characterized by extreme sun sensitivity and additional features of frequent skin cancer in XP or XPCS and varied congenital abnormalities in XPCS [17–19]. The critical role of XPB in NER is in local DNA melting at repair sites. XPB binds to distorted DNA helices and utilizes a noncanonical mechanism to exert local DNA strand separation around nucleotide lesions, which is mediated by conformational changes upon ATP hydrolysis, but is not dependent on its helicase activity [20, 21]. This initial XPB-mediated DNA opening allows the XPD helicase to anchor and unwind the DNA, promoting the subsequent steps NER [22].

From a therapeutic perspective, small molecule inhibitors have been identified that target XPB, thus preventing NER, but also impacting transcription. The natural product triptolide covalently binds XPB and inhibits its ATPase activity [23]. While triptolide has been shown to sensitize cancer cells to cisplatin and at low doses may preferentially inhibit NER [24, 25], it also leads to global transcription inhibition by inducing degradation of RNAPII [26]. A water soluble derivative of triptolide, minnelide, has advanced to phase I–II clinical trials (Table 5), with a focus in pancreatic cancer, though therapeutic effects are thought to be driven primarily by transcriptional modulation of cancer-associated genes, rather than NER inhibition [27]. Spironolactone, a diuretic and cardiovascular drug, has also been shown in several studies to degrade XPB and inhibit DDR and pro-survival signaling, with less effects on global transcription [28–30]. A series of preclinical combination studies with spironolactone showcased its potential efficacy as an effective repurposed drug for cancer treatment. For example, the combination of spironolactone with either cisplatin or carboplatin was shown to increase the therapeutic response in bladder cancer models [31]. In another study, it was shown to increase the efficacy of LP-184, an alkylator, in glioblastoma xenografts with decreased tumor relapse [32]. The combination of LP-184 and spironolactone is undergoing clinical investigation in a phase I study in patients with advanced solid tumors (NCT05933265, Table 5).

### Xeroderma pigmentosum D (XPD/ERCC2)

XPD/ERCC2 is a 5′–3′ helicase that acts in conjunction with XPB helicase in the NER pathway (reviewed in [16]). Germline defects similarly can cause XP, TTD, or XPCS. XPD has an Fe-S cluster which is postulated to interact directly with the DNA substrate and act as a DNA damage sensor [33–35] and has been shown to be important for its helicase activity. Like XPB, XPD belongs to the SF2 superfamily of helicases and is a part of the TFIIH transcription complex. As mentioned above, the ATPase activity of XPB initially unwinds the DNA at damaged sites, allowing XPD to bind to the ssDNA and translocate along the DNA strand. This movement halts at the lesion leading to the recruitment of the nucleases, XPG, and XPF-ERCC1 to carry out dual incisions on either side of the lesion for subsequent removal. Mutations in the C-terminal domain of XPD are seen in patients with TTD (R658H and R722W) and XP (R683W). Importantly, these sites lie within the domain of XPD interacting domain with p44, a subunit of the TFIIH transcription complex and regulatory partner of XPD [36]. As a result, the helicase activity of XPD is inhibited leading to a dysfunctional repair response to bulky DNA adducts [37]. Additionally, the interaction of XPD with the cyclin-dependent kinase (CDK)-activating kinase complex via its ARCH domain has been shown to reduce its helicase activity, suggesting an intricate regulation of XPD by its interactors [38].

Interestingly, while XPD participates in both NER and transcription initiation, its helicase and ATPase activity are essential only for NER and are not required for transcription [39]. There have been no reported studies on therapeutic targeting of XPD to our knowledge; however, the specificity of XPD helicase activity requirement towards NER offers an attractive therapeutic avenue for future exploration.

### Cockayne Syndrome B (CSB/ERCC6)

CSB/ERCC6 is a member of the SNF2/SWI2 family of the SF2 helicase superfamily and contains a central ATPase domain within which lies 7 conserved helicase motifs (reviewed in [40]). Germline mutations in CSB (and CSA) are the underlying cause in Cockayne syndrome (CS) which manifests as growth deficiency and severe neurological problems [41]. Like other SNF/SWI family members, CSB does not display DNA strand separating activity but behaves as an ATP-dependent dsDNA translocase, modifying chromatin to allow repair factors to access the damaged site within the DNA [42]. In the TC-NER pathway, CSB binds to stalled RNAPII at DNA lesions, an interaction that requires CSB ATPase activity to expose a C-terminal region important for chromatin association [43]. This RNAPII-CSB complex then recruits CSA and subsequent NER repair factors [44, 45]. Separately, CSB-mediated chromatin remodeling has been shown to be important for TC-NER, possibly by removing barriers for other repair factors to enable efficient repair [46].

Interestingly, CSB participates in additional DNA repair pathways via its ATPase activity. The chromatin remodeling activity of CSB appears to play a role in the resolution of DNA double strand breaks (DSBs), wherein CSB suppresses nonhomologous end joining (NHEJ) and shifts the equilibrium towards homologous recombination repair (HRR) of DSBs in cells within the S/G2 phase [47]. Specifically, upon recruitment to DSBs, CSB evicts histones from chromatin thereby limiting accumulation of the NHEJ factor RIF1 while promoting BRCA1 accumulation [48]. CSB furthermore directly acts with BRCA1 to promote the end resection step of HRR [49]. CSB has also been shown to participate in BER through its recruitment of XRCC1 protein to sites of 8-oxoguanine lesions, as well as through stimulation of APE-1 endonuclease activity, which occurs independent of ATP hydrolysis [50, 51]. Finally, in DNA SSB-R, particularly in actively transcribed DNA regions, CSB is recruited by PARP1 and PARP2 and promotes recruitment of downstream repair factors as well as dissociation of PARP from chromatin to facilitate repair [50–53].

Consistent with the function of CSB in multiple DDR pathways, its loss has been shown to sensitize cells to various DNA damaging agents, including cisplatin, oxidizing agents, and PARP inhibitors [52, 54, 55]. CSB has thus been proposed as a therapeutic oncologic target, particularly as it was shown to be overexpressed in cancer cells relative to normal cells, though no small molecule inhibitors have been reported as of yet [56–58].

## DSB-R-associated helicases

When cells are exposed to radiation or radiomimetic drugs, the sugar phosphate backbone of DNA is attacked by radiolytic radicals that form DNA single strand breaks (SSBs) [59]. The formation of these breaks on both strands of DNA gives rise to the more potent DNA DSBs [60]. DSBs can also form due to collisions between DNA replication machinery and various impediments, such as DNA secondary structures, bulky adducts, interstrand crosslinks (ICLs), DNA–protein crosslinks, and even transcription complexes [61, 62]. DSBs disrupt the normal processes of DNA and can result in chromosomal translocations leading to cancer and other diseases associated with chromosomal instability [63]. In mammalian cells, there are multiple pathways for DSB-R, of which the HRR and NHEJ pathways constitute the two major pathways [64]. HRR is an error-free pathway that uses homologous DNA sequences to resolve DSBs, and as a result, is most prevalent during the S/G2 cell phase of the cell cycle [65]. NHEJ is an error-prone pathway that modifies DSB ends and ligates the two ends leading to small nucleotide insertions or deletions within the sequence, and may be utilized throughout the cell cycle [66]. Alternative end joining (alt-EJ) pathways, more specifically Pol theta-mediated end joining (TMEJ) or microhomology-mediated end joining (MMEJ), comprise a third mechanism of DSB-R that begins with an end resection step followed by ligation of DSB ends but requires microhomologies, often resulting in large insertions or deletions or leading to chromosomal translocations [67]. Lastly, single-strand annealing (SSA) is another homology-dependent DSB-R pathway which uses tandem repeats at DSB ends for repair, but unlike HRR, is highly error-prone and leads to large deletions due to extensive end resection [68]. These pathways involve a myriad of proteins, some of which are DNA helicases that exhibit DNA unwinding and/or ATP-dependent translocase activities. Here, we summarize some of the important helicases participating in these pathways (Table 2).

**Table 2.** Helicases involved in DSB-R pathways

Helicase	Classification	Biochemical specificity	DDR function(s)	Genetic syndromes	Reported inhibitors	Additional DNA repair pathway involvement
DNA2	SF1	5′–3′ helicase Bidirectional nuclease	Participates in end-resection step of HRR Participates in mitochondrial LP-BER Promotes EXO1-independent MMR	MPD, RTS-like, MDS	NSC-15765, Anticancer agent 168, NSC-105808	ICL-R, BER, MMR
HELB	SF1	5′–3′ ssDNA translocase	Limits DNA2-BLM- and EXO1-mediated end resection Promotes RAD51-mediated strand exchange Displaces RPA from ssDNA in vitro	-	-	-
HELQ	SF2 (Ski2-like family)	3′–5′ helicase	Promotes strand annealing in multiple DSB-R pathways Promotes DSB end resection by EXO1 Participates in ICL-R independent of FA pathway May upregulate NER	-	-	ICL-R, NER
RECQL1	SF2 (RecQ family)	3′–5′ helicase	Promotes replication fork restart Modulates Ku70/80 to facilitate NHEJ Participates in novel sub-pathway of LP-BER	RECON syndrome	-	ICL-R, BER
BLM	SF2 (RecQ family)	3′–5′ helicase	Participates in end-resection during HRR Resolves DNA repair intermediates to limit inappropriate recombination Interacts with FA proteins for ICL-R Suppresses MMR-induced cell death	BS	ML216, AO/854	ICL-R, BER
WRN	SF2 (RecQ family)	3′–5′ helicase 3′–5′ exonuclease	Regulates DSB-R pathway choice among HRR, NHEJ, and alt-EJ Resolves secondary structures during HRR and at expanded TA-dinucleotide repeats Promotes BER through stimulating activities of Pol β, NEIL1, and FEN1	WS	NSC-19630, NSC-617145, GSK_WRN3, GSK_WRN4, GSK4418959, HRO761, VVD-133214, MOM-341, NTX-452	ICL-R, BER
RECQL4	SF2 (RecQ family)	3′–5′ helicase	Promotes end resection during HRR Stabilizes NHEJ machinery Stimulates strand annealing for NHEJ and alt-EJ Possible role in FA-independent ICL-R Promotes multiple steps of BER	RTS, RAPADILINO syndrome, BGS	-	ICL-R, BER
RECQL5	SF2 (RecQ family)	3′–5′ helicase	Disrupts RAD51 presynaptic filaments and limits inappropriate recombination Promotes RAD52-mediated SDSA Plays a role in repair of ICLs and oxidative DNA damage	-	1,3,4-oxadiazole derivative	ICL-R, BER
DDX11	SF2 (Fe-S cluster family)	5′–3′ helicase	Participates in HRR and facilitates end resection Promotes ICL-R in parallel to FA pathway	WBS	-	ICL-R
RTEL1	SF2 (Fe-S cluster family)	5′–3′ helicase	Regulates HRR by suppressing D-loop intermediates Protects stalled replication forks	DC, HHS	-	ICL-R
PIF1	SF1B	5′–3′ helicase	Promotes DSB end resection of DNA containing G4 quadruplexes Promotes break-induced replication (BIR) pathway	-	4-phenylthiazol-2-amine derivatives	-
MCM8/9	AAA+ family	3′–5′ helicase	Promotes DSB end-resection mediated by MRN Promotes HRR repair synthesis downstream of RAD51 Participates in MMR by stimulating MLH1 recruitment	POF, LLS	-	ICL-R, MMR

DNA2, DNA replication helicase/nuclease 2; HELB, DNA helicase B; HELQ, Helicase, POLQ-like; RECQL1, RecQ-like helicase; BLM, Bloom syndrome helicase; WRN, Werner syndrome helicase; RECQL4, RecQ-like helicase 4; RECQL5, RecQ-like helicase 5; DDX11, DEAD/H-box helicase 11; RTEL1, Regulator of telomere elongation 1; PIF1, Petite integration factor 1; MCM8/9, Minichromosome maintenance 8/9; SF1, Superfamily 1; SF2, Superfamily 2; AAA+, Triple-A ATPase; HRR, Homologous recombination repair; LP-BER, Long-patch base excision repair; MMR, Mismatch repair; ICL-R, Interstrand crosslink repair; FA, Fanconi anemia; NER, Nucleotide excision repair; NHEJ, Nonhomologous end joining; alt-EJ, Alternative end-joining; BER, Base excision repair; SDSA, Synthesis-dependent strand annealing; MPD, Microcephalic primordial dwarfism; RTS-like, Rothmund–Thomson like syndrome; MDS, Mitochondrial DNA depletion syndrome; RECON, RECql One; BS, Bloom Syndrome; WS, Werner Syndrome; RTS, Rothmund–Thomson syndrome; BGS, Baller–Gerold syndrome; WBS, Warsaw Breakage syndrome; DC, Dyskeratosis congenita; HHS, Hoyeraal–Hreidarsson syndrome; POF, Premature ovarian failure; LLS, Lynch-like syndrome

### DNA replication helicase/nuclease 2 (DNA2)

DNA2 is an SF1 helicase predominantly involved in DNA end resection, a pivotal early step in HRR-mediated repair of DNA DSBs wherein a 3′-end ssDNA overhang is generated that subsequently facilitates the recruitment of HRR proteins (reviewed in [69, 70]). The first pair of HRR factors that are recruited to DNA DSBs are the MRN (MRE11-RAD50-NBS1) complex and its cofactor CtIP, which generate short 3′-ssDNA overhangs, and in turn recruit downstream factors, EXO1 and DNA2-BLM, for long-range end resection [71]. DNA2 harbors ATP-dependent 5′–3′ helicase and bidirectional nuclease activities *in vitro* [72]. Clinically, germline mutations in DNA2 have been associated with multiple human disorders including microcephalic primordial dwarfism (MPD) [73], Rothmund–Thomson like syndrome (RTS-like) [74], and mitochondrial DNA depletion syndrome which can be associated with multisystem dysfunction or epilepsy [75, 76]. Interestingly, it is the nuclease rather than the helicase activity of DNA2 that has predominant significance in end resection during HRR. In this context, a recent study by Pinto *et al.*, showed that DNA2 was able to unwind kilobases of DNA *in vitro* but only in the absence of its nuclease activity, possibly because the nuclease activity hinders the helicase activity by competing for the same DNA substrate [77]. Instead, DNA2 interacts with both BLM and WRN, and the helicase activities of these proteins are closely coordinated with DNA2-mediated end resection (discussed further in subsequent sections) [78].

Multiple studies have shown DNA2 to be present both in the nucleus and the mitochondria although it contains no nuclear localization signal but does harbor a mitochondrial localization signal [79, 80]. The role of DNA2 in the mitochondrial DNA maintenance has been attributed to the resolution of flap intermediates formed during Okazaki fragment maturation as well as long patch base excision repair (LP-BER), in conjunction with the FEN1 nuclease. Notably, the repair of mitochondrial DNA harboring oxidative damage was impaired when DNA2 was depleted in HeLa cells [80]. DNA2 has also been shown to play a role in EXO1-independent MMR [81].

Due to its aforementioned roles, DNA2 has been implicated in providing cancer cells the ability to resist replication stress and DNA damage induced by chemotherapy drugs. DNA2 expression is also elevated in tumors compared to corresponding normal tissue across many types of cancers [82, 83]. Several small molecule inhibitors of DNA2 nuclease and helicase activities, including some nitroquinoline carboxylic acid derivatives (NSC-1576 or “Compound C5” and Anticancer agent 168 or “Compound d16”) and a quinolonedione compound (NSC-105808) have been identified which sensitize cancer cells to a range of chemotherapeutics, as well as demonstrate synergy with PARP inhibitors [82–84].

### DNA helicase B (HELB/HDHB)

HELB is a member of the SF1 helicases with an important but relatively understudied role in maintaining genome integrity (reviewed in [85]). It possesses a central helicase domain that is involved in its binding to ssDNA, 5′–3′ ssDNA translocase activity, and critical interaction with Replication Protein A (RPA) [86]. HELB has minimal helicase activity *in vitro* and while this can be enhanced in the presence of high external force, its relevance in a cellular setting remains to be elucidated [87]. The DDR role of HELB was initially suggested by its accumulation on chromatin in response to genotoxic stress and its requirement for recovery from replication stress induced by UV irradiation, camptothecin, or hydroxyurea. Mechanistically, RPA has been shown to recruit HELB to DNA DSB sites, where it limits DNA2-BLM- and EXO1-mediated end resection via its 5′–3′ translocase activity in an RPA-dependent manner [88]. In the absence of HELB, unrestricted end resection may promote a BRCA1-independent pathway of DSB-R, as loss of HELB results in PARP inhibitor resistance in BRCA1-deficient cells. HELB has also been shown to stimulate RAD51-mediated strand exchange, specifically by promoting heteroduplex extension, suggesting an additional distinct role in HRR [89]. More recently, HELB was shown to displace RPA from ssDNA using its 5′–3′ translocase activity *in vitro*, and it is postulated that this displacement of RPA is important for subsequent DNA transactions [87].

HELB was recently identified as a novel susceptibility gene for ovarian cancer [90]. However, given the still limited understanding of the different functions of HELB in DDR, it remains to be determined whether targeting HELB will emerge as clinically useful. Nevertheless, the loss of HELB as a mechanism of PARP inhibitor resistance presents potential therapeutic significance worthy of further study.

### Helicase, POLQ-like (HELQ)

HELQ is an ATP-dependent 3′–5′ helicase belonging to the Ski2-like subfamily of SF2 helicases and is involved predominantly in DNA replication, recombination, and repair (reviewed in [91]). HELQ binds to and translocates on ssDNA, unwinds duplex DNA structures, and stimulates DNA strand annealing [92–94]. Using recombinant proteins, the DNA unwinding activity of HELQ has been observed to be stimulated by RAD51 while its strand annealing activity is promoted by RPA [92]. Consistent with its ability to promote strand annealing, knockdown of HELQ is detrimental to multiple sub-pathways of DSB-R which involve the annealing of complementary ssDNA strands, including SSA, MMEJ, TMEJ, and synthesis-dependent strand annealing (SDSA) [92, 95]. HELQ may impinge upon other steps in DSB-R in addition to strand annealing, as a recent study showed that HELQ promotes DSB end resection by EXO1 via its binding to ssDNA and helicase activity [96].

HELQ is additionally implicated in ICL-R as its depletion leads to sensitivity to ICL agents like MMC [97]. Interestingly, the role of HELQ in ICL-R appears independent from the FA pathway as dual depletion of HELQ and FANCD2 yielded increased sensitivity relative to their individual depletion. Whether this role of HELQ in ICL-R is directly tied to its functions in DSB-R remains untested. Of note, HELQ has been shown to limit replication fork degradation by stabilizing RAD51 at reversed forks, independent of its helicase activity, a function which could also be relevant in ICL-R [96]. Finally, HELQ may also promote NER via upregulation of proteins involved in the NER pathway, though the mechanism underlying this observation remains unknown [98].

HELQ polymorphisms have been associated with risk of various cancers, and preclinical studies have suggested that HELQ may play a tumor suppressor role as depletion of HELQ leads to markers of tumorigenesis *in vitro* [91, 99, 100]. HELQ does not appear to be consistently up- or down-regulated in cancers compared to corresponding normal tissues [91]. However, overexpression of HELQ in ovarian cancer has been associated with platinum drug resistance and poor prognosis [101, 102] and depletion of HELQ confers ICL sensitivity, suggesting a potential therapeutic avenue for which additional validation is needed.

### RECQ helicases

The human RECQ family consists of highly conserved 3′–5′ helicases that are involved in a wide range of cellular processes and play a crucial role in maintaining genome integrity (reviewed in [103]). There are five known human RECQ family helicases: RECQL1, BLM, WRN, RECQL4, and RECQL5. In addition to critical roles in replication and DSB-R, the RECQ helicases also display an overlapping phenotype of interaction with BER-mediated repair of oxidative damage or DNA SSBs. In this section, we will review each of these helicases individually and summarize some of their pivotal roles in the DDR pathways.

#### RECQL1/RECQ1

RECQL1/RECQ1 is the most highly expressed member within the RECQ family and plays an integral role in DNA replication and repair, particularly both DSB-R and BER. Biallelic mutations in RECQL1 have been recently implicated as the underlying cause of the genome instability disorder, RECON (RECql One) syndrome, characterized by short stature, photosensitivity, and an increased sensitivity to DNA damaging agents [104]. Depletion of RECQL1 increases the sensitivity of cancer cells to numerous DNA damaging agents including camptothecin, gemcitabine, temozolomide, and ionizing radiation [104–107]. Suggesting a potential role in HRR, RECQL1 displays Holliday junction branch migration and strand annealing activities *in vitro*, associates with RAD51 in cells, and protects cells from increased sister chromatid exchanges [107–109]. However, the impact of RECQL1 on HRR of DNA DSBs induced by I-SceI measured by reporter assay was found to be minimal [110]. More recently, RECQL1 has been implicated in replication fork restart following genotoxin-induced replication stress, a process counter-regulated by PARP1 [111–113], likely underlying the sensitivity of RECQL1-deficient cells to diverse genotoxic stressors. RECQL1 has also been shown to interact with the Ku70/Ku80 heterodimer *in vitro* and unwind Ku-bound DNA, and the absence of RECQL1 decreased NHEJ efficiency in cell free extracts, suggesting another role in DSB-R [114].

Oxidatively damaged bases are an underlying cause of aging and are normally resolved within cells via the BER pathway which comprises of the short-patch and long-patch (LP) repair pathways. Cells depleted of RECQL1 are more sensitive to oxidative base damage and notably show an increase in PARP activity upon oxidative stress [110]. The interaction of RECQL1 with PARP was further elucidated in a study by Woodrick *et al.*, wherein RECQL1 was shown to participate in a newly discovered sub-pathway of long-patch BER (LP-BER) involving the formation of 9-nt gap 5′ to the DNA lesion in a RECQL1 helicase- and ERCC1-XPF endonuclease-dependent manner. Furthermore, RECQL1-depleted cells showed a preference for PARP1-mediated single nucleotide BER as compared to LP-BER, suggesting that RECQL1 regulates PARP1 BER activity and BER pathway choice [115].

RECQL1 has been found to be overexpressed in several different human cancers including glioblastoma, low-grade glioma, and multiple myeloma [116–118]. As discussed previously, depletion of RECQL1 sensitizes cells to numerous DNA damaging agents and has also been shown to enhance cell sensitivity to both PARP and PARG inhibitors [117, 119]. RECQL1 small interfering RNA (siRNA) therapies have been shown to have activity in mouse xenograft tumors, alone and in combination with DNA damaging chemotherapies, suggesting the potential for future development of RECQL1 inhibitors [120].

#### Bloom syndrome protein (BLM)

BLM is a member of the RecQ family of 3′–5′ DNA helicases and is implicated in the rare autosomal recessive disorder, Bloom syndrome, characterized by growth retardation, photosensitivity, and high susceptibility to cancer [121, 122]. BLM-deficient cells display genomic instability marked by high levels of sister chromatid exchanges, which requires BLM helicase activity for correction [123, 124].

BLM has a multifaceted role in HRR of DNA DSBs with both pro- and antirecombinogenic activities having been described. First, BLM is a critical factor in end resection during HRR, in which it both utilizes its helicase function in conjunction with DNA2 nuclease function to resect dsDNA and stimulates EXO1 independent of its helicase activity [71, 125]. As the extent of DNA end resection is a key factor driving DSB-R choice, BLM promotes HRR over NHEJ, as well as limits alt-EJ pathways [126]. Paradoxically, BLM has also been shown to exhibit antirecombinogenic properties wherein it disrupts the association of RAD51 with ssDNA and dissolves D-loops, the latter of which is regulated by a BLM interacting complex of TopoIIIα-RMI1-RMI2, thus allowing regulation of a balance between stabilization and dissolution of D-loops [127, 128]. Finally, in the downstream steps of HRR, BLM in complex with TopoIIIα-RMI1-RMI2 promotes dissolution of double Holliday junctions to form noncrossover products [129–131].

BLM’s helicase activity is also instrumental in unwinding DNA secondary structures including G-quadruplexes and R-loops *in vitro* and suppresses recombination in transcribed genes [132–134]. BLM is also required for efficient ICL-R mediated by the FA pathway, with numerous studies demonstrating physical and functional interactions with FA proteins [135–139]. In particular, BLM interacts closely with FANCM and FANCJ, helicases involved in ICL-R, which are discussed further in future sections [136, 139]. The role of BLM in BER is less established, but BLM does interact with and stimulate the activity of FEN1, an endonuclease involved in LP-BER [140]. Finally, BLM is also recruited by the MMR system [141] to sites of alkylation damage where it suppresses MMR-induced cell death [142].

In addition to the significant cancer predisposition seen in Bloom syndrome, BLM has been shown to be overexpressed or mutated in multiple cancer types including prostate cancer [143], colon cancer [144], and glioma [145]. Additionally, BLM has been shown to be involved in the alternative lengthening of telomeres (ALT) mechanism prevalent in many cancers underscoring the magnitude of BLM’s importance in carcinogenesis [146]. In this regard, several BLM inhibitors including AO/854 and ML216 have been shown to be effective either as a monotherapy or in combination with chemotherapeutic agents such as cisplatin in cell and xenograft models [147–150]. New quinazolinone and quinolone derivatives have also been shown to inhibit BLM and synergize with PARP inhibition [151]. Recently, a novel BLM inhibitor displaying highly selective allosteric inhibition *in vitro* has also been reported [152].

#### Werner syndrome protein (WRN)

WRN is a 3′–5′ DNA helicase and is a part of the RecQ family of helicases [153]. Autosomal recessive mutations in the WRN protein underlie Werner syndrome, a premature aging syndrome associated with a high cancer predisposition [154–156]. WRN has been shown to unwind a wide range of DNA substrates, including 3′-tailed duplex DNA, Holiday junction intermediates, bubble structures, and G-quadruplex DNA [157]. Additionally, WRN is the only member within the RecQ family to have a 3′–5′ exonuclease activity [158, 159]. WRN plays pivotal roles in different DNA repair pathways which mainly include HRR and NHEJ for repairing DNA DSBs and BER for repairing oxidatively damaged bases.

In DNA DSB-R, WRN has been shown to interact closely with NHEJ and alt-EJ factors. WRN is recruited to DSBs by Ku70/80 and cooperates with XRCC4-DNA ligase IV in end processing [160, 161]. Both the helicase and nuclease activities of WRN are critical in stimulating classical NHEJ [162]. Additionally, WRN inhibits recruitment of CtIP and MRE11 to sites of DSB ends in a nonenzymatic manner, thereby inhibiting DNA end resection and favoring NHEJ over alt-EJ repair [162]. Conversely, WRN has been shown to promote long-range end resection in cooperation with DNA2 in the HRR pathway [71, 78]. To explain these divergent functions, WRN has been reported to act as a DSB-R “pathway switch” wherein CDK1-mediated phosphorylation of WRN (S1133) in S/G2-phase cells stimulate long-range end resection, promoting HRR over NHEJ [163].

WRN deficiency results in accumulation of cellular oxidative DNA damage, consistent with the premature aging phenotype of Werner syndrome and a role for WRN in BER. In a study by Harrigan *et al.*, WRN was shown to unwind BER substrates *in vitro* as well as interact with and promote DNA pol β strand displacement synthesis, which is involved LP-BER repair pathway [164]. Subsequently, WRN exonuclease activity was shown to cooperate with pol β in the repair BER lesions with a 3′ mismatch, and depletion of WRN resulted in impaired cellular LP-BER [165]. In addition, independent of its helicase activity, WRN stimulates NEIL1 glycosylase activity on oxidative DNA lesions as well as FEN1 endonuclease activity [166, 167].

WRN has emerged as an important potential therapeutic target and several small molecule inhibitors of WRN have been developed. Initially, small molecule inhibitors (NSC-19630, NSC-617145) demonstrated inhibition of cell proliferation and induction of DNA damage in a WRN-dependent manner as well as synergy with PARP inhibitors, topoisomerase poisons, or the interstrand crosslinking agent mitomycin C, the latter being observed particularly in FANCD2-deficient cells [168, 169]. However, these compounds were later identified to be limited by nonspecific protein precipitation [170]. In 2019, it was discovered that cancers with high microsatellite instability (MSI-H) are highly dependent on WRN helicase function and that depletion of WRN selectively kills MSI-H cancer cells [171–174]. Mechanistically, WRN helicase activity is critical for resolution of secondary DNA structures formed within TA-dinucleotide repeats which undergo large scale expansions in MSI-H cells [175]. Since then, numerous additional WRN inhibitors have been developed and tested primarily in MSI-H cancers. Some promising compounds include GSK_WRN3 and GSK_WRN4 [176] which are covalent inhibitors that bind WRN Cys727 and inhibit its helicase activity, HRO761 which is a noncovalent allosteric inhibitor targeting the helicase domain [177], and VVD-133214 which is a covalent allosteric inhibitor also targeting Csy727 [178]. All three compounds selectively inhibited growth of MSI-H cancer cell and patient-derived xenograft models, and phase I trials have been initiated with HRO761 and VVD-133214 in MSI-H or MMR-deficient cancer. More recently, several additional novel small molecule WRN inhibitors, including GSK4418959, NTX-452, and MOMA-341 [179–181], have also entered early stage clinical trials, either as a monotherapy or in combination with immunotherapy or chemotherapy (Table 5).

#### RECQL4/RECQ4

RECQL4 is a 3′–5′ DNA helicase involved in DNA replication fork progression, the maintenance of mitochondrial and telomere integrity, and multiple DNA repair pathways including DSB-R and BER [182–185]. Mutations in RECQL4 [186] have been heavily implicated in the human disorders, RTS [187], RAPADILINO syndrome [188], and Baller–Gerold syndrome [189]. These syndromes are characterized by overlapping manifestations including skeletal abnormalities, short stature, and cancer predisposition.

Biochemically, RECQL4 displays weak DNA unwinding activity, which is counteracted *in vitro* by strong ssDNA annealing activity, and it selectively recognizes Holliday junctions [190–192]. In a study by Singh *et al.*, RTS fibroblasts showed moderate sensitivity to IR with increased accumulation of the γ-H2AX and 53BP1, and laser microirradiated cells showed early accumulation of exogenously expressed RECQL4 to these sites [193]. RECQL4 has subsequently been shown to impact multiple pathways of DNA DSB-R. Regarding its role in HRR, RECQL4 helicase activity was shown to mediate 5′ end resection at laser-induced DSBs, and inactivation of the helicase domain impaired homology-dependent DSB-R [194]. RECQL4 also interacts with Ku70/80 heterodimer, and RECQL4-depleted cells demonstrate decreased NHEJ both *in vitro* and *in vivo* [195]. NHEJ stimulation by RECQL4 was later shown to be mediated by DNA-PKcs phosphorylation of RECQL4, leading to stabilization of NHEJ machinery at DSBs [196]. More recently, a critical role for interaction with PARP1 has been identified, wherein PARP1 is required for recruitment of RECQL4 to DSBs and PARG-mediated dePARylation of RECQL4 stimulates its strand annealing activity to promote both NHEJ and alt-EJ [197]. Furthermore, RECQL4 has been shown to modulate DSB-R pathway choice between HRR and NHEJ, in a process mediated by the phosphorylation and ubiquitination of RECQL4 in S/G2 phases, which promotes the accumulation of RECQL4 at DSBs to drive end resection and stimulate HRR in these phases of the cell cycle [198].

Apart from its role in DSB-R, RECQL4 has also been postulated to play a role in FA-independent ICL repair, based largely on data derived with the yeast homologue HRQ1 [199, 200]. RECQL4 depletion in U2OS cells was shown to increase cellular sensitivity to the crosslinking agent cisplatin [201]. Finally, like RECQL1 and WRN, RECQL4 has also been shown to be involved in BER of oxidative DNA damage through its regulation of the strand displacement activity of Pol β, the nuclease activities of FEN1 and APE1, and the glycosylase activity of OGG1 [202, 203].

Like many of its other family members, RECQL4 is overexpressed in a variety of cancer types, including prostate cancer and breast cancer, and has been correlated with advanced stages, aggressive tumors, and poor outcomes [201, 204, 205]. Suppression of RECQL4 in these settings can limit tumor cell growth and sensitize cells to chemotherapy *in vitro*, supporting its potential as a therapeutic target.

#### RECQL5/RECQ5

RECQL5 is the fifth member of the RECQ family and exists in three isoforms, RECQL5α, RECQL5β, and RECQL5γ, out of which only RECQLβ (referred to as RECQL5 from hereon) shows nuclear localization [206]. RECQL5 displays significant strand annealing activity on DNA duplexes, with this activity harbored in a unique C terminal domain, distinct from its conserved 3′–5′ helicase domain [207, 208]. RECQL5 is recruited to sites of DNA DSBs in a MRE11-dependent manner, where it interacts with RAD51 and disrupts RAD51 presynaptic filaments [209–212]. In a study by Paliwal *et al.*, this antirecombinase activity of RECQL5 was shown to promote RAD52-mediated SDSA during HRR of DNA DSBs, favoring noncrossover repair products over crossover products [213]. In this manner, RECQL5 is believed to regulate HRR and limit inappropriate recombination. Like other RecQ helicases, RECQL5 also interacts with PAR and PARP1, which regulates its helicase and strand annealing activities in a complex manner [214].

RECQL5 also plays a role in a variety of other pathways important for genome stability. RECQL5 is recruited to psoralen ICLs via its KIX domain and is involved in the early unhooking step of ICL repair [215]. Numerous reports have also implicated RECQL5 in preventing transcription-associated genome stability as well as stabilizing stalled replication forks [215–219]. Finally, knockdown of RECQL5 led to an increase in oxidative DNA lesions, both endogenously and after treatment with oxidizing agent, and RECQL5 was found to accumulate at laser induced SSBs, suggesting a role of RECQL5 in BER [220].

There are no well-established germline syndromes associated with RECQL5, but RECQL5 mutations have been implicated in risk of multiple cancers including osteosarcoma [221, 222], breast cancer [223, 224], and colorectal cancer [225]. Amplification or overexpression of RECQL5 has also been identified as a common phenomenon across multiple cancer types and has been associated with poor prognosis [226–228]. Recently, pharmacologic inhibition of RECQL5 by a 1,3,4-oxadiazole derivative (compound 4a) was shown to specifically kill RECQL5-expressing cancer cells, re-sensitize cisplatin-resistant breast cancer cells, and reduce growth in preclinical breast cancer xenograft models [229]. In addition, combination of this inhibitor with PARP inhibition led to synergistic killing of breast cancer cells via HRR inhibition and concomitant NHEJ hyperactivation [230].

### DEAD/H-box helicase 11 (DDX11)

DDX11 is an ATP-dependent, Fe-S cluster containing 5′–3′ helicase belonging to the SF2 DNA helicases (reviewed in [231]). Mutations in DDX11 are the underlying cause of the rare cohesinopathy, Warsaw Breakage syndrome, which is characterized clinically by impaired growth, hearing loss, and increased risk of cancer [232, 233]. Early biochemical studies with DDX11 showed that a 5′ ssDNA region is required for DDX11’s unwinding activity and that it was able to unwind a wide range of DNA substrates including forked duplex, 5′-flaps, and G-quadruplexes, among others [234, 235]. DDX11 participates in the DDR by promoting DSB-R via homologous recombination. Specifically, DDX11 has been shown to facilitate end resection and allow loading of RPA and RAD51 onto ssDNA, acting nonepistatically to both BRCA1 and BRCA2 [236]. Furthermore, using avian cells, Abe *et al.* demonstrated that DDX11 acts collaterally with the FA pathway in ICL-R wherein *ddx11/fancc* double mutants exhibited enhanced sensitivity to ICL-inducing agents [237].

DDX11 expression is upregulated in many cancers, including hepatocellular carcinoma, melanoma, and renal cell carcinoma, and has been shown in some cases to be essential for disease progression or survival [238–241]. Depletion of DDX11 was shown to impart cellular sensitivity to cisplatin, bleomycin, and PARP inhibitors, suggesting DDX11 as an exploitable target [236, 242, 243]. Moreover, loss of DDX11 can resensitize BRCA1-deficient cells with acquired drug resistance to PARP inhibitors [236], further highlighting its potential role as a therapeutic drug target, though DDX11 inhibitors have not yet been reported.

### Regulator of telomere elongation 1

Regulator of telomere elongation 1 (RTEL1) is a 5′–3′ DNA helicase belonging to the subfamily of Fe-S helicases within the SF2 helicases which also includes XPD, DDX11, and FANCJ [244, 245]. Germline biallelic pathogenic RTEL1 alterations are associated with dyskeratosis congenita (DC) which is characterized by dystrophic nails, abnormal skin pigmentation, oral leukoplakia, bone marrow failure, and cancer predisposition, and with Hoyeraal–Hreidarsson syndrome, a severe form of DC additionally characterized by cerebellar hypoplasia [246]. RTEL1 is important for genomic integrity largely through its involvement in telomere maintenance [247]. Specifically, RTEL1 facilitates telomeric DNA replication by dismantling T-loops and resolving G-quadruplex structures [248].

RTEL1 additionally contributes to genomic integrity through its regulation of DNA replication and repair [249]. Early studies showed that siRNA-mediated knockdown of RTEL1 led to > 3-fold increase in HRR in an I-SceI DSB assay in SW480/SN3 cells and that RTEL1 disrupts D-loop intermediates *in vitro*, underscoring the importance of RTEL1 in suppressing HRR [250]. More recently, RTEL1 helicase activity was shown to counteract RAD51-mediated homologous recombination and fork reversal at stalled replication forks, thereby protecting cells from replication stress [251]. RTEL1 additionally protects cells from replication stress by suppressing R-loop formation and limits transcription-replication conflicts [252, 253]. Finally, depletion of RTEL1 results in increased sensitivity to ICL-inducing agents, though its precise role in ICL-R remains unclear [254].

RTEL1 functions as a tumor suppressor gene as mutations have been associated with increased risk of high grade gliomas and other cancers [255]. At the same time, RTEL1 has been shown to be upregulated in gastrointestinal cancers and may promote carcinogenesis, likely through its roles in telomere maintenance and facilitating replication [256–258]. RTEL1 depletion in this setting inhibited tumor growth and metastasis, suggesting its potential as another therapeutic drug target.

### Petite integration frequency 1 helicase (PIF1)

PIF1 is an ATP-dependent 5′–3′ helicase belonging to the SF1B family of helicases and homologue of the *Escherichia coli*RecD helicase (reviewed in [259]). Varied functions of PIF1 are important for genomic integrity and primarily include resolution of G-quadruplexes and R-loops, telomere maintenance, and DNA synthesis under replication stress [259–262]. With respect to its role in DSB-R, PIF1 was shown to localize to sites of DSBs, and PIF1 knockdown impaired HRR with no effect on NHEJ, suggesting that PIF1 shifts the equilibrium of DNA DSB-R towards HRR. Mechanistically, PIF1 was shown to be involved in long-range DNA end resection and importantly, required to resect G quadruplex-forming DNA ends, suggesting a specific role in the resection of DNA with complex secondary structure [263]. PIF1 additionally plays a role in BIR, a pathway of homology-mediated DSB-R requiring long-track replication [264, 265].

Elevated PIF1 levels are observed in many cancer types whereas expression is low in differentiated normal tissues and it is dispensable in Pif1-deficient mice, suggesting it may be promising candidate for therapeutic intervention. Although no small molecule inhibitors of PIF1 have been tested clinically, 4-phenylthiazol-2-amine derivatives have been shown to inhibit PIF1’s helicase activity *in vitro* and warrant future studies [266].

### Minichromosome maintenance 8/9 (MCM8/9)

The eukaryotic minichromosome maintenance (MCM) proteins [267] are comprised of the dynamic hexameric MCM2-7 complex [268, 269] and the MCM8/9 heterodimer [270]. MCM2-7 has been shown to be involved exclusively in the initiation and elongation phases of eukaryotic replication [271, 272]. Conversely, MCM8/9, paralogs of MCM2-7 consisting of a MCM domain and an AAA + ATPase domain and exhibiting 3′–5′ helicase activity, play an important role in DNA repair processes and mutations have been associated with premature ovarian failure, chromosomal instability, and cancer predisposition [273–275].

MCM8/9 were originally shown to be required for HRR associated with ICLs and DNA DSBs using chicken DT40 cells [276]. Human cells depleted of MCM8/9 similarly exhibited hypersensitivity to ICLs and failed to recruit RAD51 to DNA DSB sites [277]. A subsequent study by Lee *et al.* showed that the MCM8/9 complex promotes DSB end resection by the MRN complex [278]. MCM8/9 also acts downstream of RAD51 via an association with RPA-associated factor MCM8IP (or HROB) and promotes repair synthesis in parallel to the HELQ helicase [279, 280].

Apart from its role in HRR, MCM8/9 has also been shown to be involved in MMR [281]. MCM8/9 was identified in complex with numerous MMR proteins in cell extracts, including MSH2 and MLH1. Mechanistically, MSH2 was found to recruit MCM8/9 to chromatin, which in turn stimulated MLH1 chromatin binding. Functionally, loss of MCM9 helicase function led to reduced MMR activity in a reporter assay and onset of microsatellite instability [281]. Reflecting this role in MMR, germline biallelic MCM8 variants have been associated with early-onset Lynch-like syndrome [282].

MCM8 has been shown to be overexpressed in certain cancers compared to corresponding normal tissue, including cholangiocarcinoma, bladder cancer, and gastric cancer, and associated with poor prognosis [283–285]. Depletion of MCM8 in models of these cancers was successful in impairing tumorigenesis. In addition, depletion or knockout of the MCM8/9 complex has been shown to sensitize tumor cells, but not untransformed human cells, to cisplatin and the PARP inhibitor, olaparib [286, 287].

## ICL-R-associated helicases

ICLs are one of the most deleterious DNA lesions affecting both strands of DNA and inhibiting major DNA processes like replication and transcription [288]. While some ICLs are detected and repaired in nonreplicating cells, the predominant pathways of ICL-R occur during S phase upon collision with a replication fork. Replication-coupled ICL-R is a highly intricate process which involves the overarching Fanconi anemia (FA) pathway in conjunction with NER, translesion synthesis, and HRR factors, altogether employing an interplay of >30 cellular proteins. Briefly, when a replication complex encounters an ICL in duplex DNA, replication stalls and a DDR is triggered leading to the recruitment of the FA pathway machinery. Within this pathway, FANCM along with FAAP24 and MHF (MHF1 and MHF2), acts as a “triad” that binds the stalled fork DNA structure and recruits the other FA core complex members (comprising of eight FA proteins and two FA-associated proteins). This further leads to the monoubiquitination of FANCD2 and FANCI proteins which subsequently promotes the recruitment of nucleases, polymerases, and ligases that unhook the ICL and complete the repair process [289, 290]. Mutations in >20 genes involved in ICL-R underlie FA, a rare genetic disorder characterized by dysfunctional or impaired DNA repair leading to bone marrow failure, physical abnormalities, and a strong susceptibility to cancer. Most of the helicases involved in HRR (discussed previously) have also been implicated in ICL-R given the requirement for HRR as the final step of FA-mediated ICL-R. In addition to these, FANCM and FANCJ are the two main helicases within the FA repair pathway itself and that are predominantly involved in the repair of ICLs (Table 3).

**Table 3.** Helicases involved in ICL-R*

Helicase	Classification	Biochemical specificity	DDR function(s)	Genetic syndromes	Reported inhibitors	Additional DNA repair pathway involvement
FANCM	SF2	DNA branchpoint translocase	Binds stalled forks and recruits downstream FA proteins to initiate repair of ICLs Promotes replication traverse of ICLs Suppresses ALT	Cancer predisposition syndrome	-	-
FANCJ	SF2 (Fe-S cluster family)	5′–3′ helicase/ATPase	Participates in ICL-R downstream and/or in parallel to FANCD2 Promotes end resection during HRR May facilitate processing of ICL-R intermediates Interacts with MMR proteins and suppresses microsatellite instability	FA	-	DSB-R, MMR
*Many helicases involved in DSB-R are also implicated in ICL-R						

FANCM, Fanconi anemia complementation group M; FANCJ, Fanconi anemia complementation group J; SF2, Superfamily 2; ICL, Interstrand crosslink; FANCD2, Fanconi anemia complementation group D2; HRR, Homologous recombination repair; FA, Fanconi anemia; DSB-R, Double strand break repair

### Fanconi anemia complementation group M (FANCM)

FANCM is a DEAH-helicase [291] belonging to the SF2 superfamily of helicases. Biallelic loss of function mutations in FANCM are associated with cancer predisposition, but not the congenital malformations or bone marrow failure of FA [292, 293]. FANCM ATP-dependent enzyme activity is required for cellular resistance to ICLs, though its role in FANCD2 monoubiquitination occurs independent of its ATPase activity, suggesting multiple functions for FANCM in ICL-R [294]. Interestingly, as seen above with some helicases, studies with human FANCM detected no canonical helicase activity during its involvement in FA-ICL repair [294]. Rather, FANCM possesses a specialized “branchpoint translocase” activity (harbored within the N-terminal helicase domain of FANCM) wherein it drives the directional migration of Holliday junctions or other branched structures in an ATP-dependent manner [295–297]. This activity is distinct from helicase unwinding in that it requires homologous sequences both upstream and downstream of the branchpoint. FANCM branchpoint translocation is critical for fork reversal at sites of stalled replication due to ICLs, an important step in FA-mediated ICL repair. In addition, a study by Huang *et al.* [298] observed that FANCM, dependent on its translocase activity, is able to facilitate the replication machinery to “traverse” replication forks stalled due to unrepaired ICLs, allowing DNA replication and synthesis to proceed in the presence of a lingering ICL which is then removed by downstream post-replication processes. FANCM interacts directly with the BLM complex, and BLM helicase activity promotes FANCM recruitment to stalled forks, activation of the FANCD2-FANCI complex, and efficient traverse and repair of DNA ICLs [136]. In addition, FANCM is phosphorylated by ATR which also promotes its ICL repair and replication traversal activities [299].

Outside of ICL repair, FANCM translocase activity is involved in additional genome maintenance roles including replication fork protection under replication stress, R-loop resolution, and, of potential therapeutic importance, suppression of ALT [300–302]. ALT, a process which maintains telomere length independent of telomerase, is utilized in ∼15% of cancers. Importantly, depletion of FANCM leads to excessive ALT activity which ultimately generates lethal replication stress at telomeres [301]. Thus, targeting FANCM, as has recently been demonstrated with antisense oligonucleotides [303] may offer an opportunity for synthetic lethal treatment of ALT-positive cancers.

### Fanconi anemia complementation group J (FANCJ)

FANCJ/BRCA1-associated C-terminal helicase 1 (BACH1)/BRCA1-interacting protein 1 (BRIP1) is a DNA helicase belonging to the SF2 superfamily, with a conserved Fe-S domain in the N-terminal region. As the name suggests, FANCJ interacts with the BRCA1 protein and as such was initially identified as a potential DSB-R factor [304]. Subsequently, mutations in FANCJ were identified in Fanconi anemia complementation group J (FANCJ) and were associated with chromosomal instability, increased sister chromatid exchanges, and hypersensitivity to DNA interstrand crosslinking agents [139, 305–307]. FANCJ possesses both 5′–3′ helicase and DNA-dependent ATPase activities. *In vitro*, FANCJ displays preferential unwinding of forked duplex DNA substrates. Additionally, FANCJ resolves 5′-flap substrates and displays 5′–3′ branch migration activity, which represent species of D-loops formed during HR, but shows no activity toward Holliday junctions [308, 309]. FANCJ interacts with RPA in the presence of DNA damage, and this interaction enhances the unwinding activity of FANCJ [310].

In the context of ICL-R, FANCJ-deficient cells display intact FANCD2 monoubiquitination, suggesting that FANCJ acts downstream of the FA core complex [311]. Conversely, nonmonoubiquitinated FANCD2 interacts with FANCJ and FANCD2 foci formation is FANCJ-dependent [312, 313], suggesting a possible role of FANCJ in priming FANCD2 for subsequent activation. FANCJ has also been proposed to facilitate processing of downstream ICL-R intermediates for subsequent HRR repair [308]. In addition, FANCJ has been shown to be required for DNA end resection during HR repair via its interaction with CtIP nuclease and its helicase activity [314]. Interestingly, FANCJ interacts directly with the MMR protein MLH1, and disruption of this interaction induces ICL sensitivity, which appears to be mediated by an MSH2-dependent DDR response as depletion of MSH2 corrects this sensitivity [315, 316]. FANCJ was also shown to be recruited to sites of UV damage in an NER- and MMR-dependent fashion [317].

Outside of its direct role in classical DNA repair pathways, FANCJ helicase activity has been implicated in several other mechanisms relating to genome maintenance including resolution of G-quadruplexes, replication restart after fork stalling, processing of DNA–protein crosslinks, and suppression of microsatellite instability [318–322]. In response to replication stress, FANCJ interacts physically and functionally with BLM helicase, with FANCJ playing a critical role in BLM protein stability [139]. More recently, a functional interaction between FANCJ and PARP1 has been elucidated in which FANCJ, via its MLH1 interaction and ability to process G-quadruplex structures bound by MSH2 and PARP1, is required for PARP1 activation during replication [323]. Interestingly, while FANCJ loss induces an HR-deficiency phenotype, it does not lead to PARP inhibitor sensitivity; however, loss of FANCJ is synthetic lethal with BRCA1-deficiency [323].

Clinically, in addition to germline loss of FANCJ underlying a rare subtype of FA, FANCJ/BRIP1 variants are also associated with hereditary breast and ovarian cancer syndrome [324]. FANCJ expression is also dysregulated across many cancer types [325]. As loss of FANCJ sensitizes to agents that induce ICLs or DSBs, the various alterations of FANCJ in cancer may give rise to either resistance of sensitivity to DNA damaging agents. As of yet, no specific inhibitors of FANCJ have been reported, though would be of interest for study in BRCA1-deficient cancers.

## DRR-associated helicases

### Activating signal co-integrator complex 3 (ASCC3)

DNA base alkylation lesions can arise intracellularly via metabolites such as S-adenosyl methionine (SAM) or can be induced externally by alkylating agents like temozolomide or methyl methanesulfonate. DRR enzymes catalyze direct removal of specific alkyl lesions without requiring excision of the damaged base. O-6-methylguanine-DNA methyltransferase (MGMT) forms one component of DRR and specifically resolves O^6^-methylguananine (O^6^-MeG) lesions [326]. The family of human alkylated DNA repair protein B (AlkB) homologs are the second component of DRR and comprise of nine nucleic acid demethylases with unique substrate specificities (reviewed in [327]). Two of the best characterized ALKBH proteins, ALKBH2 and ALKBH3, are involved in the removal of 1-methyladenine (1-MeA) and 3-methylcytosine (3-MeC) from DNA [328]. Other alkyl lesions, including N^7^-MeG and N^3^-MeA, are processed by the BER pathway [329].

In a study by Dango *et al.*, ALKBH3 was found to interact with the Activating Signal Co-integrator Complex (ASCC), specifically with largest subunit of the complex, ASCC3. ASCC3 is an SF2 family member with 3′–5′ helicase activity and was found to promote DRR mediated by ALKBH3 [330] (Table 4). Interestingly, although ALKBH3 displays a preference for repair of lesions within ssDNA, in conjunction with the unwinding activity of ASCC3, it was able to repair alkylated dsDNA. Furthermore, depletion of either ALKBH3 or ASCC3 in prostate cancer cells led to accumulation of 3-MeC and DNA damage signaling, along with reduced cell proliferation and xenograft tumor growth. Whether additional helicases interface with other ALKBH enzymes for repair of alkylation damage remains to be determined.

**Table 4.** Helicases involved in DRR

Helicase	Classification	Biochemical specificity	DDR function(s)	Genetic syndromes	Reported inhibitors	Additional DNA repair pathway involvement
ASCC3	SF2 (Ski2-like family)	3′–5′ helicase	Unwinds DNA for ALKBH3-mediated DRR	Neuromuscular syndrome	-	-

ASCC3, Activating signal co-integrator 1 complex subunit 3; SF2, Superfamily 2; ALKBH3, AlkB homolog 3

Clinically, biallelic variants in ASCC3 have been implicated in a neuromuscular syndrome characterized by developmental and neurological defects [331, 332]. Additionally, somatic cancer mutations have been shown to cluster at the interface of the ASCC2-ASCC3 interaction, suggesting the possibility that decreased efficiency of alkylation repair may predispose to malignancy [333]. Finally, while there is limited data on ASCC3 expression in cancer, ALKBH3 is highly overexpressed in multiple cancer types, [334, 335] and could potentially serve as biomarker for tumors dependent on ASCC3-ALKBH3 mediated repair.

## Perspectives and conclusion

As detailed in the above sections, helicases are integral components of DDR pathways and prevent genomic instability via facilitating and ensuring proper DNA repair. Over the past several decades, key advances have been made in understanding the detailed mechanisms of helicase function and their implications in cellular pathways, including DDR. This sum of this research has highlighted the complexity of not only individual DDR helicases, but also the interactions among different helicases and within the broader DDR network. While DDR helicases contain structurally similar catalytic domains across families and superfamilies, they are highly specialized in their biochemical functions which are in turn critical for differentiating cellular functions. Apart from DNA unwinding activity, other catalytic activities (e.g. exonuclease or strand annealing activity) as well as noncatalytic activities (e.g. recruitment or stimulation of other DDR factors) often are just as important to DDR helicase biological function and further differentiate their cellular roles. In some cases, *in vitro* biochemical function has been closely tied to cellular activity in a specific repair step, but in many cases the implications of different biochemical specificity are still being parsed out. On a higher level, DDR helicases comprise a complex yet specialized network of “DNA untanglers”. Multiple DDR helicases often display apparently redundant functions within a DDR pathway. At the same time, individual DDR helicases frequently impinge upon multiple different DDR pathways. Finally, at the highest level, DDR helicases have complex interactions with other DDR factors which closely modulate their activity. For example, DDR helicases can promote or inhibit DDR pathways as a result of protein-protein interactions with or post-translational protein modifications induced by other DDR factors. While a full detailing of these interactions was beyond the scope of this review, this complex network is crucial for the ability of DDR helicases to affect their roles in maintaining genomic integrity and remains to be fully elucidated.

As the understanding of DDR helicases has advanced, they are now emerging as an important class of potential therapeutic targets. Fundamentally, DDR helicases are connected to human disease via the syndromes that result upon germline loss of function of the majority of the known DDR helicases. These disorders are often characterized by developmental abnormalities, premature aging, and predisposition to cancer, underscoring the importance of DDR helicases in normal maintenance of genome integrity, faithful cell division, and prevention of mutagenesis. More unique features of these DDR helicase genetic disorders are understood in relation to their specific DDR-related or DDR-adjacent protein functions, though many of these ties remain poorly understood. Beyond the inherited genetic disorders, variants in DDR helicases have increasingly been identified as important cancer-predisposing alterations. Conversely, gain of function, overexpression, or dysregulation of DDR helicases (most predominantly seen with the RECQ family helicases as well others involved in DSB-R) has been observed across many human cancer types. Excessive helicase activity likely serves to protect cancer cells from the extra stressors of rapid cell division, counteract inherent genomic instability of cancer, and confer resistance to DNA damaging chemotherapeutics.

In the existing research investigating therapeutic opportunities targeting DDR helicases, several themes emerge. First, for tumors arising in the setting of DDR helicase defects, an intrinsic cellular vulnerability exists which may frequently lead to chemosensitization to a specific DNA damage that is susceptible to a repair pathway that is no longer intact. For example, cells with loss of FANCJ or FANCM, as well as many DSB-R helicases, display hypersensitivity to ICL inducing agents [294, 306]. Second, for cancers with overexpression or dysregulation of DDR helicase, it is commonly seen that tumor cells are addicted to this elevated helicase activity and that tumor growth can be hindered by helicase depletion or inhibition, as seen for example with DNA2, RTEL1, and RECQ family helicases, among others [83, 120, 205, 258]. DDR helicase small molecule inhibitors, if highly specific, could be utilized in this setting with potential for a therapeutic index relative to normal tissue. Third, drug combination strategies, involving the combination of a DDR helicase inhibitor with a DNA damaging agent or another DDR inhibitor have in many cases been shown to display synergistic activity in *in vitro* and preclinical tumor models. For instance, inhibition of XPB was shown synergize with the alkylator LP-184 in GBM and inhibitors of DNA2, BLM, WRN, or RECQL5 were all shown to synergize with PARP inhibitors [32, 84, 151, 169, 230]. Fourth, a deep understanding of the mechanisms of certain helicases has led to identification of novel synthetic lethal interactions in specific cellular settings highly relevant to cancer, exemplified by the synthetic lethality of WRN loss in MSI-H tumors or FANCM loss in ALT tumors [171–174, 301]. These latter therapeutic opportunities have garnered considerable excitement, with WRN inhibitors being investigated in multiple human clinical trials of MSI-H cancers. Finally, although beyond the scope of this review, reactivation of gene function in germline genetic disorders using CRISPR-Cas9-based gene editing is an area of intense research. Recent progress has been made in FA with a human clinical trial demonstrating bone marrow failure reversal in FA patients with mutations in *FANCA* following gene correction of autologous hematopoietic cells [336], and a similar strategy may be applicable to *FANCJ* mutations as well. Application of this technology to DDR helicase disorders more generally will be challenging given their multisystem manifestations, but does offer an exciting area for investigation.

Despite the promising applications of DDR helicase inhibitors, several challenges have been identified in the development of such inhibitors. Notably, many of the early inhibitors of BLM and WRN helicases were subsequently shown to have nonspecific inhibitory effects, such as induction of protein aggregation or precipitation or binding of DNA, leading to false-positive results in screening assays [170]. Reported inhibitors targeting other DDR helicases largely lack extensive biophysical validation and may suffer from similar issues. Helicases themselves present several challenges to drug targeting including their high conformational dynamics which complicates structure-based drug design, and the conservation of active sites among helicases which renders targeting of a specific helicase more difficult [337]. In addition, given the importance of noncanonical helicase activities to their overall function, inhibitors targeting the helicase domain may in some cases not have the desired or full therapeutic effect. Nevertheless, recent studies have made advances in the development of helicase inhibitors by utilizing mass spectrometry-based chemoproteomics to identify covalent inhibitors or using “scout-fragments” to identify druggable allosteric sites [178, 338]. Several highly specific well-validated inhibitors have now been described, establishing the feasibility of helicase inhibitor development. Thus far, nonspecific inhibitors of XPB and novel specific inhibitors of WRN are the main DDR helicase targeting agents to enter human trials (Table 5).

**Table 5.** Completed and ongoing oncologic clinical trials

Helicase	Inhibitor	Clinical trial	Trial stage	Indication/cancer type	Concurrent therapy	Human safety information
XPB	Minnelide	NCT01927965	I	Advanced gastrointestinal tumors	-	Gastrointestinal toxicity (nausea, vomiting, abdominal pain), neutropenia, cerebellar dysfunction [346, 347]
		NCT03117920	II	Refractory pancreatic cancer	-	
		NCT03129139	I	Advanced solid tumors	Monotherapy or in combination with nab-paclitaxel	
		NCT04896073	I	Advanced pancreatic cancer	-	
		NCT05166616	I	Advanced EGFR-mutant NSCLC	Osimertinib	
		NCT05566834	I	Advanced gastric cancer	Monotherapy or in combination with paclitaxel	
		NCT05557851	Ib	Metastatic pancreatic cancer	Gemcitabine + nab-paclitaxel	
	Spironolactone	NCT05933265	I/II	Advanced solid tumors	LP-184	Hyperkalemia and other electrolyte imbalances, gynecomastia
WRN	GSK4418959	SYLVER (NCT06710847)	I/II	MSI-high or MMR-deficient solid tumors	Monotherapy or in combination with PD-1 inhibitor	Being evaluated
	HRO761	NCT05838768	I/Ib	MSI-high or MMR-deficient advanced solid tumors	Monotherapy or in combination with pembrolizumab or irinotecan	Being evaluated
	VVD-133214 / RO7589831	NCT06004245	I	MSI-high or MMR-deficient advanced solid tumors	Monotherapy or in combination with pembrolizumab	Being evaluated
	MOMA-341	NCT06974110	I	MSI-high or MMR-deficient advanced solid tumors	Monotherapy or in combination with immunotherapy or irinotecan	Being evaluated
	NDI-219216 / NTX-452	NCT06898450	I/II	Advanced solid tumors	-	Being evaluated

XPB, Xeroderma pigmentosum B; WRN, Werner syndrome helicase; MSI, microsatellite instability; MMR, mismatch repair

To expand the therapeutic potential of DDR helicases, we envision future advances arising in both the mechanistic and therapeutic arenas. Mechanistically, much remains to be learned about the biological functions of certain understudied helicases (e.g. HELB, PIF3), the redundancies and distinctions of different helicase functions, and the complex regulation of helicases within DDR pathways. Recent advances in super resolution microscopy, single-molecule analysis techniques, and artificial intelligence prediction tools are expected to help decipher these functions. Therapeutically, while several promising synthetic lethal strategies have been described and are under active investigation, there are other reported co-dependencies, such as those seen with combined loss of BRCA1 and FANCJ [323] or combined loss of RECQL1 and PARG activity [197], which merit further investigation. Additional studies using advanced screening modalities, for example, through CRISPR/Cas9-based screens, are likely to discover additional novel co-dependencies. On the drug innovation side, additional ways to target helicases apart from small molecule inhibitors, for example through use of PROTACS, have also been reported with promising results [339]. New methods applied to other enzymatic targets, such as protease engineering [340] or antisense oligonucleotides [341] may also prove fruitful for targeting DDR helicases. Altogether, DNA helicases within the critical DDR network offer significant opportunities for future biological and therapeutic studies.

## Data Availability

No new data were generated or analyzed for this review.
